# A modified oddball paradigm for investigation of neural correlates of attention: a simultaneous ERP–fMRI study

**DOI:** 10.1007/s10334-013-0374-7

**Published:** 2013-03-17

**Authors:** Mateusz Rusiniak, Monika Lewandowska, Tomasz Wolak, Agnieszka Pluta, Rafał Milner, Małgorzata Ganc, Andrzej Włodarczyk, Andrzej Senderski, Lech Śliwa, Henryk Skarżyński

**Affiliations:** 1 World Hearing Center of The Institute of Physiology and Pathology of Hearing, Mokra 17 Str., 05-830 Nadarzyn, Poland; 2 Children’s Memorial Health Institute, Al.Dzieci Polskich 20 Str., 04-730 Warsaw, Poland

**Keywords:** Oddball, fMRI, EEG, P300, Event-related potentials, Attention

## Abstract

**Introduction:**

The objective of the presented study was to develop and evaluate a P300 experimental protocol for simultaneous registration of event-related potentials (ERPs) and functional MRI (fMRI) data with continuous imaging. It may be useful for investigating attention and working memory processes in specific populations, such as children and neuropsychiatric patients.

**Materials and methods:**

Eleven children were investigated with simultaneous ERP–fMRI. To fulfill requirements of both BOLD and electroencephalographic signal registration, a modified oddball task was used. To verify the ERP–fMRI protocol we also performed a study outside the scanner using a typical two-stimuli oddball paradigm.

**Results:**

Localization of the P300 component of ERPs partially corresponded with fMRI results in the frontal and parietal brain regions. FMRI activations were found in: middle frontal gyrus, insula, SMA, parietal lobule, thalamus, and cerebellum. Our modified oddball task provided ERP–fMRI results with high level of significance (EEG SNR = 35, fMRI *p* < 0.05–Bonf.). ERPs obtained in the scanner were comparable with those registered outside the scanner, although some differences in the amplitude were noticed, mainly in the N100 component.

**Conclusion:**

In our opinion the presented paradigm may be successfully applied for simultaneous ERP–fMRI registration of neural correlates of attention in vulnerable populations.

## Introduction

Neural correlates of auditory attention in both normal and pathological states are still a topic of debate. The most common techniques used to investigate the brain mechanisms underlying attentional processes are event-related potentials (ERP) and functional MRI (fMRI).

The ERP technique measures synchronized synaptic activity of neural networks with high temporal resolution (tens to hundreds of milliseconds). P300, a positive component which peaks about 300 ms after the stimulus onset [1, 2], is “endogenous”, i.e. depends strongly on cognitive processes involved in a given task [3]. It was first described by Sutton and coworkers [4] as a positive deflection that appeared under conditions of uncertainty about the nature of the upcoming stimulus. P300 is often investigated with an “oddball” paradigm, in which an occasionally occurring deviant stimulus has to be detected in a train of frequent, standard stimuli. In response to deviants, P300 is usually observed in the parietocentral area of the scalp [5]. The topography on the scalp allows a distinction to be made between a more frontal early P3a component and a more parietally pronounced P3b component. The P3a component reflects automatic novelty detection and P3b is associated with volitional deviant detection [1, 5]. A significant limitation of ERP studies is that, due to volume conduction and the inverse problem, the generators of EEG activity cannot be reliably inferred on the basis of topographical distribution alone. Thus, to label the brain structures of interest, it is better to combine the source localization methods based on the EEG with other brain imaging methods, e.g. functional MRI.

fMRI measures brain activity by detecting task-associated changes in blood flow, i.e. the hemodynamic response. One common fMRI technique described by Ogawa et al. [6] uses the blood-oxygen-level-dependent (BOLD) contrast to study cognitive processes with high spatial resolution (ca. 3 mm).

Numerous fMRI studies show that during performance of various auditory attention tasks the fronto-parietal network is activated [7–9]. Additionally, fMRI responses to tasks that typically evoke the electrophysiological P300 component appear to involve a distributed network including the supramarginal gyrus, temporo-occipital and superior temporal regions, the supplementary motor area (SMA), as well as the cingulate gyrus, insula, thalamus and cerebellum [10–16]. In many of these studies, however, scanning did not involve the full brain volume. Because of the wide range of areas involved in the performance of an oddball task, investigation using a full brain volume fMRI might provide important data on the location of specific brain activity.

An unresolved issue in fMRI research is low temporal resolution of the hemodynamic response. Since ERP measures cortical activity with high temporal resolution, a combination of these two noninvasive methods makes it possible to map cognitive processes with both high spatial and temporal resolution.

Basic animal research using simultaneous intracortical recordings of electrical neural signals and the BOLD fMRI responses [17] has shown a clear correlation between local field potentials and the fMRI BOLD signal. Likewise, human studies conducted by Horovitz et al. [18] have revealed a strong association between the P300 and BOLD signals evoked in an oddball paradigm. They showed that manipulation of the task (e.g. the probability of the deviant event) changed both P300 and BOLD signal amplitudes. However, in this study the authors only showed how EEG and fMRI measurements can be conducted in separate sessions (not simultaneously).

Over the last decade several different approaches have been reported for studying the brain mechanisms involved in selecting the deviant sound from a group of frequent sounds (the standards), using simultaneous ERP and fMRI. Indeed, the combination is especially useful for investigating “endogenous” ERP components, such as P300, because of the multiplicity of physiological and behavioral factors that affect its properties [19].

In some studies (e.g. [20, 21]) a sparse-sampling fMRI paradigm has been used to investigate responses to auditory oddball tasks. A clear advantage of this experimental protocol is that it records the ERP data in the absence of the high background noise of the MRI scanner. However, the sparse paradigm requires a relatively long time spent inside the magnet (>30 min) and in some cases there is a need to acquire the data in two separate sessions. In our experience, this ERP–fMRI procedure is tiresome for participants, especially children, elderly people and patients with neuropsychiatric deficits. Moreover, in most cases, using the sparse paradigm does not allow the entire time course of the BOLD signal to be tracked. Auditory noise generated by the scanner during sparse data acquisition is an additional auditory stimulus that needs to be taken into consideration during analysis and interpretation of the P300 component.

In most of the existing studies simultaneously acquiring ERP and fMRI data (e.g. [21–23]) the difficulty of the auditory oddball task is not taken into consideration. In these experiments the deviant and the standard sounds differ in frequency by 150 Hz [24], 500 Hz [22, 23] or even 1,000 Hz [25]. These differences seem to be sufficient for deviant detection in healthy young adults. However, when using identical sounds for each participant, the effect of inter-individual differences in the ability to discriminate acoustic stimuli is not controlled for.

A novel promising approach to evaluating the accuracy of the ERP–fMRI auditory oddball paradigm is to compare the ERP data acquired inside and outside the MR scanner. Mulert and coworkers [23] showed a higher N100 amplitude outside the scanner than inside the scanner and yet no significant differences in P300 parameters under these two conditions. The results were interpreted in terms of the influence of the noisy environment inside the scanner on the early ERP component (N100) but not on the late P300 component.

In the presence of existing methodological concerns about the procedure of simultaneous ERP and fMRI data collection, there is a need to design an experimental protocol which: (1) requires a short time inside the MR scanner, (2) allows control of the effect of task difficulty, (3) ensures steady, continuous background scanner noise, (4) enables full head volume registration, and finally (5) provides results comparable to those obtained outside the noisy scanner environment. The aim of our study was to develop a methodology for simultaneous measurement of ERP and fMRI data during performance of an auditory oddball task. To validate this ERP–fMRI paradigm we compared ERPs measured outside and inside the MR scanner.

## Materials and methods

The ERP–fMRI study was conducted at the Bioimaging Research Center of the Institute of Physiology and Pathology of Hearing in Warsaw using a Siemens 3 T Magnetom Trio Tim MR scanner and a 64-electrode EEG Neuroscan system.

### Participants and study preparation

Eleven children (seven girls, four boys) aged from 11 to 16 years (mean age = 13 years and 6 months; SD = 1 year and 7 months) participated in our study. Children were selected as a specific population in which studies inside the scanner are especially challenging. The children were right-handed [26], had normal hearing level in both ears, i.e. below 20 dB for each of the following frequencies: 125, 250, 500, 750, 1,000, 1,500, 2,000, 4,000, and 8,000 Hz (screening audiometry), and their intellectual skills were within the normal range (verified by the Raven’s Matrices). They had no history of neuropsychiatric disorders or head injury.

Parents provided written informed consent for their children to participate in this study. The study was approved by the ethics committee at the Institute of Physiology and Pathology of Hearing and conformed to the Declaration of Helsinki for Medical Research Involving Human Subjects.

### Paradigm

Two sessions with an auditory oddball paradigm, outside and inside the scanner, were applied.

#### Stimuli

The stimuli were pure sinusoidal 200 ms tones differing in frequency. The standard tone had a frequency of 750 Hz; the deviant tone frequency was determined on the basis of a behavioral difference limen for frequency (DLF^1^) measurement conducted prior to the study. The deviant tone frequency was set higher than the standard tone frequency by adding two times the DLF value. In our participants the DLF value varied from 10 to 30 Hz, resulting in the deviant tone frequency ranging from 770 to 810 Hz (mean deviant tone frequency = 791 Hz, SD = 4.7 Hz). This procedure ensured the same level of difficulty in the frequency discrimination task for all participants.

The tones were delivered binaurally via electrostatic headphones (NordicNeuroLab, NNL) at 80 dB (C) with harmonic distortion <2 %. Before each experimental session sound quality of the headphones was validated using GRASS audiometer calibration system. The coherence between the stimulus onset and the delay generated by the applied sound stimulation system was measured. We set up a hardware configuration for appropriate testing. The three 2.2 kΩ resistors were connected in an equilateral triangle configuration. Ground, reference and signal electrodes were connected to a triangle’s apices (between resistors). The headphones used in the experiment were placed onto the resistors in such way that the headphone membrane was in the orthocenter of the triangle. When a sound was generated we were able to record the induced artifact following the stimulus marker. On this basis the delay between values was calculated with better than 1 ms precision. The delay measurement procedure allows also to verify whether any jitter appears. However, in our measurements we did not register any jitter. We observed a 76 ms delay generated by the NNL system during simultaneous ERP–fMRI and a 17 ms delay during ERP recording outside the scanner. Both these delays were subtracted from the stimulus onsets to give synchronized timing for both EEG and fMRI analyses.

#### Session inside the scanner

**Fig. 1:**
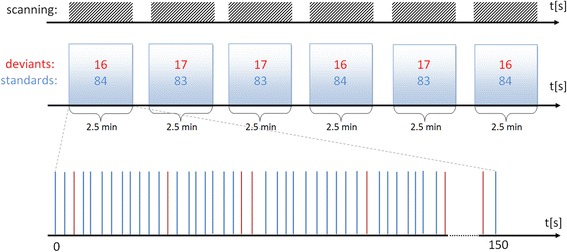
The paradigm schema. The *upper* part shows the ERP–fMRI paradigm comprising six blocks. The *lower* part presents an example sequence of deviant (*red*) and standard (*blue*) stimuli within a block. After each block the participants were asked to report orally the number of the recognized deviants. *Gaps* between the *lines* reflects the jitter used during stimulation

A modified oddball paradigm was applied to meet the requirements of both EEG and fMRI signal properties (e.g. a long BOLD signal recovery time) (see: Fig. 1). To avoid the adverse monotony due to the long examination time, the experiment was short and consisted of six similar 2.5 min blocks. The stimuli sequence was presented in a pseudo-randomized order due to some limitations connected with the physiology of the BOLD signal [27]. Each block contained 100 stimuli including either 16 or 17 deviant tones (see Fig. 1). Jitter was used to increase the surprise effect and to simplify fMRI artifact removal from the EEG recording. The rules of the presentation of deviant tones were as follows: (a) no more than two deviants in a row, (b) no more than two deviant doublets per run, (c) a minimum interval of 9 s between deviant tones.

The aim of our design of BOLD study was to investigate the difference between deviant and standard stimuli processing in the brain. Therefore, only the minimum interval between deviants was considered. Frequent (standard) stimuli presented with an inter-stimulus interval (ISI) of about 1.5 s resulted in a stable baseline condition for the fMRI contrast. The control of the ISI value for rare (deviant) stimuli ensures a well-shaped hemodynamic response function (HRF).

During each run a static, a neutral picture was presented via NordicNeuroLab OLED goggles to encourage participants to keep their eyes open. The visual presentation was used to restrain subjects from closing their eyes what might result in changing study conditions and cause unwanted brain activity.

The task was to count silently the deviant tones. After each block a short (ca. 30 s) break was introduced during which participants were asked by the experimenter how many deviants had occurred. For each participant, the response error rate was below 10 % ensuring that the subjects were accurately discriminating the sounds. The whole task took approximately 15 min.

#### Session outside the scanner

Before the ERP–fMRI measurement, one block of a similar experimental sequence (using identical sound stimuli, ISI and jitter) was performed outside the scanner. It comprised a 3 min 45 s session with an oddball paradigm involving 120 standard and 30 deviant stimuli presentations.

It is believed that the randomization method might influence the P300 potential generation, both in terms of the amplitude and the latency. Therefore, for more accurate verification of the proposed modified oddball task in the simultaneous ERP–fMRI study, the experiment outside the magnet was performed with a fully randomized paradigm.

### EEG recording

A 64-channel Neuroscan EEG system was used in the following configuration: 62 unipolar channels in 10/10 location; two bipolar channels VEOG, ECG; Fpz electrode as ground and Cpz electrode as reference. Sintered Ag/AgCl electrodes were used. Good contact between electrode and skin was assured using the Neuroscan quick-cell technology. Electrode to skin impedance was kept below 10 kΩ. Sampling rate during recording was set at 1 kHz and for each run data was recorded continuously and independently. The EEG system met all the criteria set for performing this type of recording in an MRI scanner (i.e. gradient artifacts did not saturate the amplifier).

The effect of the EEG equipment on the MRI and fMRI images was examined and no influence on fMRI data in amplitude or phase of image was found. Additionally no signal distortion in structural images occurred.

### Image acquisition

To exclude subjects with brain pathology, standard T1 and T2 sequences were applied. FMRI data were acquired continuously in 6 runs during EEG recording in a 3T scanner (Siemens Magnetom Trio TIM). A standard 12-element head matrix coil was employed for RF signal reception. The fMRI data were obtained using T2*-weighted gradient echo-planar imaging (EPI) sequence (TR 2,000 ms, TE 30 ms, FA 90°, image matrix 64 × 64, plane FOV 192 × 224 mm, iPAD = 2) with 37 ascending slices (slice thickness 3,5 mm, no gap) parallel to the axial plane. There were 78 volumes (plus three extra dummy scans) collected in the total scanning time of 2 min 36 s per run. The total number of volumes acquired during all runs was 468. No contrast agent was administered. A trigger signal was delivered by the MR scanner to the EEG recording system at each RF pulse through a synchronization device (manufactured by the Department of Electronics and Information Technology, Warsaw University of Technology) in order to guarantee an accurate matching between fMRI and EEG datasets and to improve the removal of MR artifacts from the EEG data. For each patient additional high-resolution T1-weighted images were acquired (3D MP-RAGE sequence, TR 1,900 ms, TI 900 ms, TE 2.21 ms, FA 9°, voxel size 0.9 × 0.9 × 0.9 mm, 208 sagittal-oblique slices, total scanning time 5 min) in order to provide accurate anatomical references for functional data and precise electrodes localization. This scanning sequence was acquired prior to the fMRI study, so that the patient could adapt to the MR conditions. Both techniques (EEG system and MRI scanner) were used simultaneously in order to provide ERP–fMRI registration.

### EEG signal preprocessing

Both EEG recordings, inside and outside the magnet, were analyzed in the same way, except for the removal of the artifacts induced by MRI gradients.

The EEG data was filtered and prepared in Compumedics SCAN software. We resolved the typical MRI scanning synchronization problem known in literature [19, 28] using the Neuroscan clock synchronization module which ensures synchronization between the EEG system and the scanner [19] allowing for low sampling rate.

Removal of MRI gradient artifacts was done using an in-built software function. The algorithm subtracts an adaptive template of an artifact computed using 16 surrounding artifacts from each electrode signal. The subtraction of the averaged artifact template without losing signal of interest loss was possible by using jitter and ISI which were not multiplications of TR.

Before the next step of the analysis unipolar channels were low-pass filtered using the Finite Impulse Response (FIR) filter with zero phase shift, at cutoff frequency of 50 Hz and a 24 dB/oct slope. Next, the effect of heart beat was reduced. First, the ECG bipolar channel was band-pass filtered in range 15–50 Hz with another zero phase shift FIR filter (slope 24 dB/oct). Next, automatic QRS detection was used to mark each R peak. Manual removal of incorrectly marked peaks was performed. After correcting the location of markers, balistocardiogram artifacts (BCG) [29] were averaged using a correlation based algorithm. Then a singular value decomposition (SVD) covariance matrix was computed with a retained variance of 15 %. Finally, the influence of blinking in the EEG recording was reduced. The presented methodology is congruent to that described by Otzenberger et al. [30].

The procedure of blinking artifact removal was very similar to the BCG artifact reduction. Blinks were marked automatically using a voltage threshold for the band pass filtered (1–10 Hz, zero phase shift, FIR, 12 dB/oct) from the VEOG channel. A SVD covariance matrix was constructed here with a retained variance of 30 %.

**Fig. 2:**
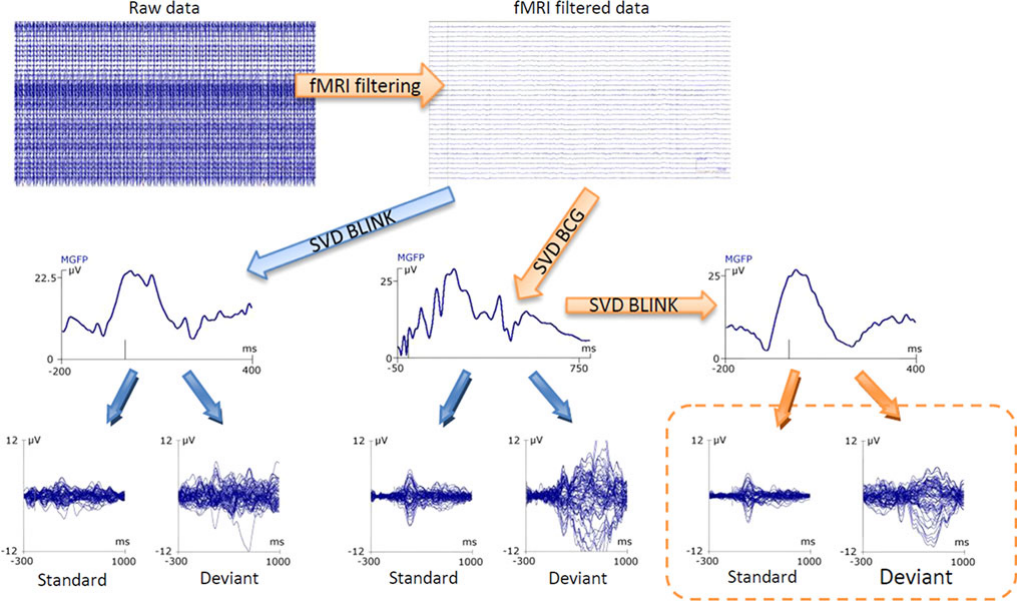
Filtering steps. Each *arrow* represents a subsequent stage of data processing. *Arrows* without a description indicate an averaging procedure. *Arrows* with labels represent filtering algorithms mentioned in the text. Final filtering steps are marked with *orange color*. The *orange dashed rectangle* includes final outcomes of the standard and deviant response obtained in the scanner for one representative subject

The abovementioned steps for artifact removal and their outcomes are presented in Fig. 2. Final filtration methods are marked in orange. After filtration, the data were considered artifact free and ready for further processing. In the next step we epoched the data according to stimulus markers in a time window from −300 to 1,000 ms. Baseline correction was then performed. The period before the stimulus onset was used as the baseline interval. Data were visually inspected for any other artifacts and averaged. Samples with substantial signal distortion were excluded from further analysis. ERP responses were filtered with a zero phase shift FIR filter with low-pass cutoff frequency of 20 Hz and 12 dB/oct slope. The data window was reduced to −100 to 800 ms because outside this interval there was no significant ERP responses detected. Finally, the data was smoothed using five-point averaging. The time map and all data statistical analyses were re-referenced to joint M1 and M2 electrodes.

### EEG statistical analysis

For statistical analysis of the EEG data a peak detection algorithm was applied to individual data. The N100 peak was marked for both standard and deviant tones. Moreover, the P300 peak was identified in response to deviant tones. The data obtained at the Fz, Cz and Pz electrodes are considered most typical in studies in which P300 is elicited [1]. However, we added to this list Cp1 and Cp2 electrodes because during registration in the magnetic field the maximal amplitude of P300 in response to deviant tones was observed at these two channels. Time windows for the N100 and P300 peaks were selected on the basis of visual inspection of individual data. The N100 peak was defined as the most negative value between 80 and 200 ms and the P300 peak was defined as the most positive value between 350 and 550 ms.

Repeated measures ANOVAs with the following factors: “Condition” (inside vs. outside the scanner) and “Electrode” (Fz vs. Cz vs. Pz vs. Cp1 vs. Cp2) were calculated separately for amplitudes and latencies of N100 and P300 and separately for deviant and standard tones to compare ERP inside and outside the scanner.

### EEG signal source analysis

Source analysis was performed with data re-referenced to the averaged electrode according to requirements of the current density (CD) analysis [31]. Pre-processed ERP responses to deviant stimuli were imported to the BESA Research 5.3.9 software. Based on visual inspection of the deviant response waveform registered during the MRI study we determined three subsequent time intervals which included the most distinguishable peaks of ERP recorded at the Cz electrode (see Fig. 4). Intervals were selected nearby inflection points in the EEG signal observed at Cz electrode. The selected intervals, 250–345 ms (interval A), 345–419 ms (interval B) and 419–560 ms (interval C) are marked in blue, red, and green, respectively, and depicted in Figs. 4 and 7. We decided to use CLARA (classical LORETA analysis recursively applied) [32] which is an iterative application of weighted LORETA images with a reduced source space in each iteration. The initial LORETA image was computed using a truncated singular value decomposition (TSVD) approach as the regularization method, with SVD cutoff at 0.005 %. For the iterations TSVD was also used with a regularization constant of 0.01 %. A 7 mm isometric voxel was used for image computation. We used a four-shell ellipsoidal head model, based on a multi-shell spherical head model described by Berg and Scherg [33]. The four homogenous shells are: the brain, the cerebro-spinal fluid (CSF), the bone, and the skin. The spherical shells are fitted to the electrode positions over an MRI head model (a Talairach-based template in this case). As bone thickness and conductivity are age-dependent, the following values recommended in the group aged 13–14 years were used: thickness—6.3 mm, relative conductivity—0.008 (CSF = 1) [33]. All the described parameters are BESA defaults and are appropriate for the presented data. CLARA computations based on time intervals provided mean images for the selected time range. On the basis of the CLARA algorithm CD time courses for the maximum values in the estimated sources were determined (Fig. 7). Interval C results in two mirrored regions therefore two CD curves were presented (for the left and right hemisphere).

### fMRI signal processing

Data analysis was performed using the Brain Voyager software (http://www.brainvoyager.com/). Single-subject analysis was performed using standard event-related procedures. Functional scans were processed in the following steps: slice scan timing correction, head motion detection and correction, removal of linear and non-linear trends, and spatial and temporal smoothing. The purpose of the pre-processing was to remove various kinds of artefacts, in order to maximize the sensitivity of the later statistical analysis. Afterwards a general linear model (GLM) and a standard HRF were fitted to the data. The functional data were coregistered with high resolution anatomical data which were also pre-processed and interpolated to isotropic resolution of 1 × 1 × 1 mm. The T1-weighted high resolution MPR volume was corrected for inhomogeneity. Next, based on the publication by Kang et al. [34] who proved that it is not necessary to use any special template for children older than 7–8 years, we performed an automatic procedure of normalization to Talairach space. Functional data were also normalized based on the same transformation, after coregistration. Each functional run was pre-processed separately and finally all 6 runs were joined by using a multi-subject GLM with predictors separated for each series. In order to test whether results obtained for individual subjects are valid at the population level, a random effects (RFX) analysis, was used to assess variability of the observed effects across subjects. In this procedure individual subjects are considered to be a representative sample of the population. If group effects are significant at the random effects level, the findings from the sample of subjects can be generalized to the population from which the subjects have been drawn. Consequently, the multi-subject RFX analysis was performed for all 11 subjects.

## Results

### Behavioral data

All participants correctly reported the number of deviants both inside and outside the scanner, committing only a few errors. There were no significant differences [*t*(1,10) = 1.03, *p* > 0.05] in the percent of missed deviants in the study inside (*M* = 4.18, SD = 1.33) and outside (*M* = 3.25, SD = 3.00) the scanner.

### fMRI data

FMRI activations were found in several brain regions typically involved in selective attention, vigilance and target detection. Brain regions implicated in the task correspond mostly to fronto-parietal areas known as the attentional network.

**Fig. 3:**
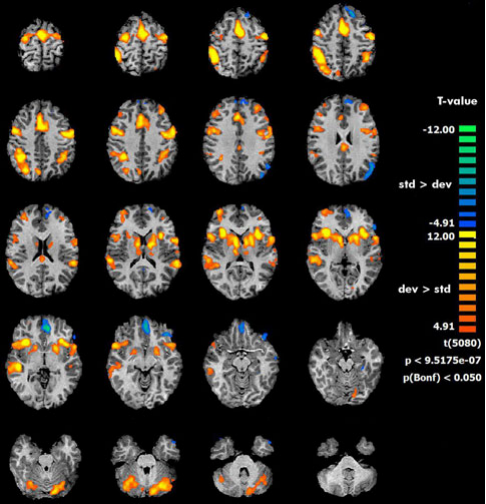
fMRI activations in deviant versus standard (*red* to *yellow*) and standard versus deviant (*blue* to *green*) contrasts, with corresponding *p* and *t* values

The group analysis for the contrast deviant versus standard tones showed activations in the following bilateral areas: insula, precentral gyrus, inferior parietal lobule, superior temporal gyrus, as well as right middle frontal gyrus, and left SMA. Additional activated regions were: the thalamus, caudate head and cerebellum (see Table 1; Fig. 3). The reversed contrast, standard versus deviant, showed increased BOLD responses in the left medial orbitofrontal cortex (OFC).

**Table 1:** fMRI results

Brain area	Cluster coordinates			Voxel peak *T*	Number of activated voxels
	*X*	*Y*	*Z*		
*Deviant versus standard*					
Frontal areas					
Insula L./BA 47	−32	20	−10	10.2	616
Insula R./BA 47	32	22	0	12.4	712
Middle frontal gyrus R.	30	−6	62	9.8	146
Precentral gyrus L./BA 6	−44	−4	36	10.1	621
Precentral gyrus R.	48	4	44	10.0	320
SMA L.	0	10	44	11.7	1,581
Parietal areas					
Inferior parietal lobule L./BA 40	−42	−46	46	8.2	186
Inferior parietal lobule R./BA 40	44	−40	56	12.3	1,174
Temporal areas					
Superior temporal gyrus L.	−56	−36	10	9.1	125
Superior temporal gyrus R.	46	−24	−10	10.3	340
Sub-cortical areas					
Caudate head	8	2	0	9.4	316
Cerebelum L.	−26	−70	−40	8.7	373
Cerebelum R.	36	−64	−38	7.9	97
Thalamus	−12	−4	6	8.8	459
*Standard versus deviant*					
Frontal areas					
Medial orbito-frontal cortex L.	−4	46	−14	7.7	75

Brain area, cluster coordinates of the maximum voxel peak, *T* statistics, and the corresponding number of activated voxels. The fMRI results were obtained with the following threshold: *t* > 7, cluster size >27

### EEG data

**Fig. 4:**
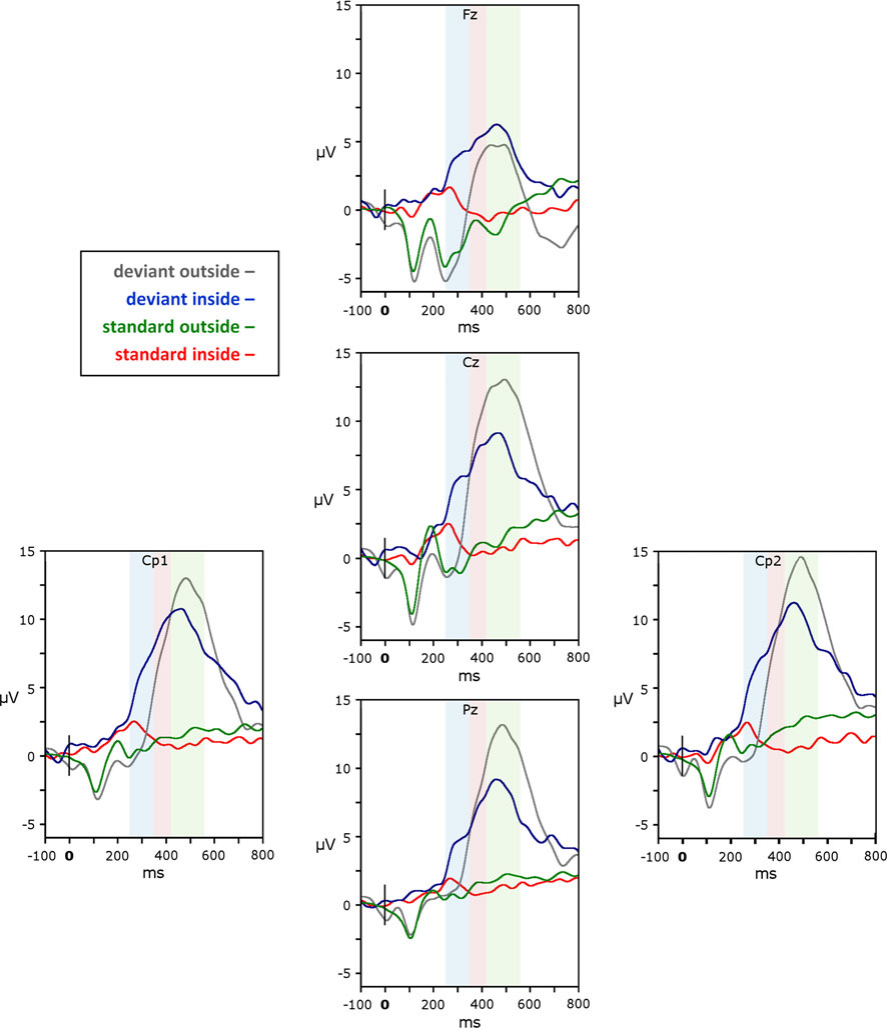
ERPs elicited at Fz, Cz, Cp1, Cp2, and Pz electrodes by deviant and standard stimuli inside and outside the magnetic scanner. *Color-shaded bars* represent intervals used for further source analysis: *blue*—interval A (250–345 ms); *red*—interval B (345–419 ms); *green*—interval C (419–560 ms)

Comparisons between the ERP inside and outside the scanner revealed significant differences in the early component (N100), which depends predominantly on sensory properties of the applied stimuli. There were some differences in the amplitude of the late endogenous P300 component, but these did not reach statistical significance (see Fig. 4).

#### N100

Repeated measures ANOVA, calculated for the amplitude of N100 component evoked by deviant tones, revealed significant main effect of “Condition” (*F*(1,10) = 11.06, *p* < 0.01) and interaction “Condition x electrode” (*F*(4,40) = 5.46, *p* < 0.05).

The amplitude of N100 component was higher outside the MR scanner (*M* = −4.64 μV, SE = 0.81) compared to inside the scanner (*M* = −1.70 μV, SE = 0.52). The post hoc comparisons for the interaction effect showed significant (*p* < 0.05) differences between N100 amplitude inside and outside the scanner at Cz (*M* = −5.22 μV, SE = 0.97 and *M* = −2.06 μV, SE = 0.77, respectively), Cp1 (*M* = −4.19 μV, SE = 0.85 and *M* = −1.40 μV, SE = 0.76, respectively), as well as Fz (*M* = −6.48 μV, SE = 0.97 and *M* = −1.50 μV, SE = 0.34) electrodes.

A similar analysis was performed for the amplitude of N100 component in response to standard stimuli. Significantly (*F*(1,10) = 33.34, *p* < 0.001) higher N100 amplitude was found outside (*M* = −3.65 μV, SE = 0.30) than inside the magnet (*M* = −0.96 μV, SE = 0.33). A significant effect of “Condition x electrode” (*F*(4,40) = 4.62, *p* < 0.05) yielded the most prominent difference between the outside and inside the scanner situation at Fz (*M* = −5.20 μV, SE = 0.59 and *M* = −1.20 μV, SE = 0.39, respectively) and at Cz (*M* = −4.42 μV, SE = 0.45 and *M* = −1.11 μV, SE = 0.42, respectively) electrodes.

There were no statistically significant differences between the two analyzed conditions in the latencies of the N100 evoked by both deviant and standard tones.

#### P300

ANOVA conducted on the amplitude of the P300 component showed no significant differences between the inside and the outside scanner conditions in both P300 amplitude (*F*(1,10) = 2.48, *p* > 0.05, *M* = 10.67 μV, SE = 1.38 and *M* = 14.02, SE = 2.01, respectively) and latency (*F* (1,10) = 1.23, *p* > 0.05, *M* = 464.04 ms, SE = 17.10 and *M* = 485.36 ms, SE = 14.50, respectively).

### EEG time course analysis

**Fig. 5:**
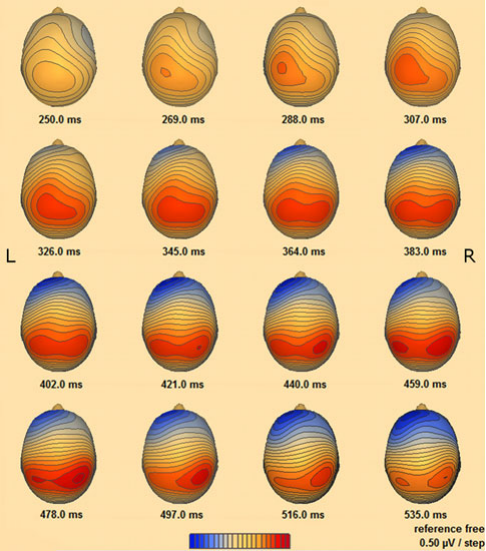
Time course of P300 potential head distribution recorded inside the scanner in time window of 250–535 ms

Figure 5 shows voltage distribution of the P300, elicited in response to deviant tones in time frame between 250 and 560 ms in subsequent 19-ms intervals (for the whole duration of the P300 waveform—see Fig. 4). The P300 distribution was found to move from the left to right parietal cortex (see Fig. 5). Interestingly, when P300 reached its maximum value it was represented in the bilateral parietal region of the brain.

### Comparisons between fMRI and EEG localization

**Fig. 6:**
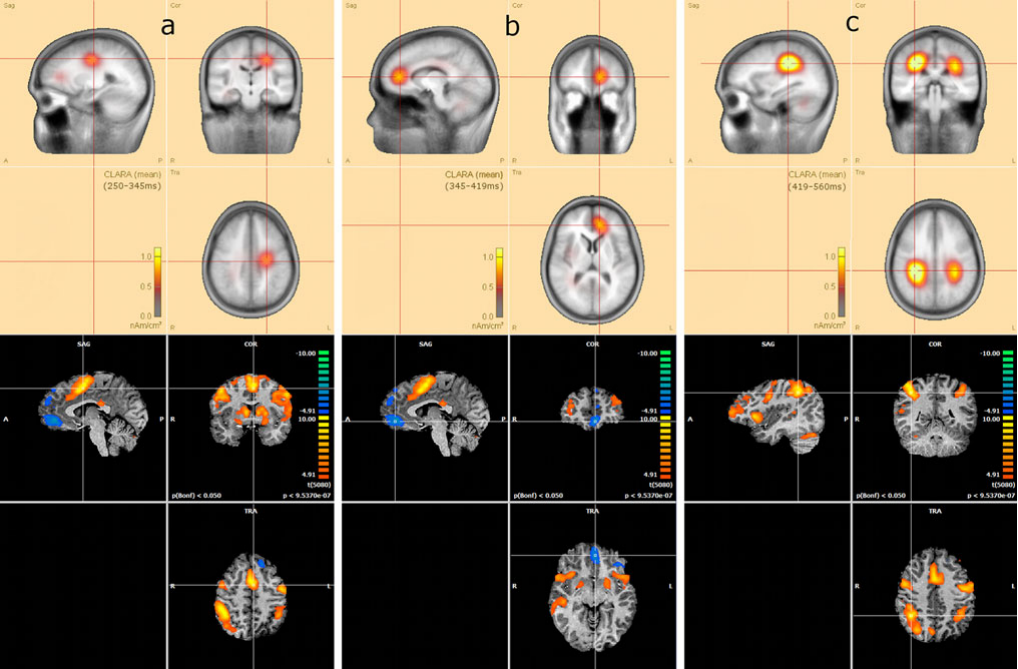
Combined results of CLARA algorithm estimation (*upper part*) and fMRI results (*lower part*); contrasts: deviant versus standard—*red* to *yellow*, standard versus deviant—*blue* to *green*. All *images* are in approximately the same Talairach coordinates. In each CLARA image the maximum mean current density value in a given interval is marked with a *crosshair*. In fMRI images a *crosshair* marks the maximum *t* value in a region most closely corresponding to ERP findings **a** at time interval A (SMA) **b** at time interval B (frontal area) **c** at time interval of C (parietal area)

As EEG and fMRI data were acquired simultaneously during an auditory task, we could integrate these results to reveal processes associated with sound discrimination. In Fig. 6 (upper row) the source estimation obtained by the CLARA algorithm independently for each interval (A, B and C) were shown. Four main areas derived from CLARA estimation (marked with red crosshairs) were matched with the fMRI results presented in the lower row in Fig. 6. In fMRI images the areas corresponding with ERP sources were marked with a crosshair. The structures obtained with this approach included: the SMA (Fig. 6a), the left medial OFC (Fig. 6b) and the inferior parietal lobule bilaterally (BA 40—Fig. 6c).

**Fig. 7:**
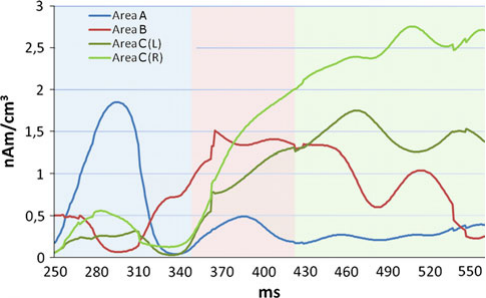
Current Density time courses in sources estimated with CLARA, showed in Fig. 6. *Blue line color* represents the CD-time course for Area A; *red*—the CD-time course for Area B; *deep green*—the CD-time course for Area C in the *right* hemisphere; *light green*—the CD-time course for Area C in the *left* hemisphere. *Color-shaded bars* represent subsequent time intervals: *blue*—interval A; *red*—interval B; *green*—interval C

The obtained ERP sources were not only prominent in generators where they reached their maxima but were also noticeable with smaller amplitudes in other time windows. Therefore, we decided to determine CD time courses (Fig. 7) for each source in a wide time interval (250–560 ms) which covered all the intervals mentioned before, i.e. A, B and C. Time-courses were produced for the maximum value in each area marked with a crosshair in Fig. 6 (upper row). The curves were named with labels corresponding to area A, B, C(L) and C(R). CLARA computed in interval C revealed two strong sources in two regions bilaterally and so two corresponding curves (L—left hemisphere, R—right hemisphere) were drawn (also for the remaining intervals). Detailed description of Fig. 7 can be found in a part of discussion entitled “Correlation between areas”.

## Discussion

The objective of the presented study was to design and verify a paradigm and complete methodology for a P300 experiment using simultaneous EEG-fMRI recording with continuous whole-brain coverage imaging. Our EEG results, obtained using a modified oddball provided outcomes congruent with those typically reported in literature [1, 5, 35]. Additionally, to better verify our paradigm we performed two study sessions: inside the MRI scanner and outside the magnet with a typical two-stimuli oddball task. Compared recordings revealed some amplitude differences described below but the properties of the P300 potential remained similar. Moreover, localization of brain activity, measured with both EEG and fMRI showed correspondence in frontal and parietal brain regions, often jointly referred to as the attention network [7]. These findings confirm the validity of our modified oddball paradigm for investigation of neural correlates of attention.

### Methodology

#### EEG data quality during fMRI scanning

In our study brain responses, especially the N100 wave, achieved during simultaneous EEG-fMRI recordings had smaller amplitudes than ERPs recorded outside the scanner. First of all, the ERP amplitude might have been affected by various noise contributions inside and outside the magnet [36]. Furthermore, although almost the same algorithms were used for data filtration in both comparable conditions (Fig. 2), the fMRI gradient removal algorithm, for obvious reasons, was only applied to EEG data recorded inside the magnet. The jitter in the ERP–fMRI was introduced to prevent any correlation between the artifact template and brain responses and, therefore, subtraction of meaningful data during the fMRI artifact removal procedure.

#### N100

When comparing data obtained inside and outside the scanner (Fig. 4), main differences in ERPs were observed in the N100 potential. Firstly, the N100 may not be apparent in EEG recordings in the magnet, due to the loud acoustic noise generated by MRI gradient coils (approx. 99 dB SPL). This was still an issue although MRI-compatible headphones were used to reduce it. The gradient auditory noise heard by the subject was around 75 dB (C). Intensity of the stimulation was set at 80 dB (C), as this level is both perceivable and neutral for the subject (i.e. it does not evoke additional eye blinking). Unfortunately, when the difference between the stimulus and the background is as minor as 5 dB, the exogenous N100 and P200 potentials might not be visible or are evoked with small amplitudes. This issue is a typical problem in simultaneous ERP–fMRI recordings [37, 38]. One probable reason of this phenomenon is a constant activation of the auditory cortex induced by the scanner noise. On the contrary, during the study outside the magnet the signal to noise ratio was approximately 40 dB (C) allowing for N100 potential elicitation. Concluding, it is worth to emphasize that the difference in the N100 potentials depends highly on the relative stimulus intensity, with smaller response amplitudes for lower relative sound levels [35].

#### P300

Although the difference in the P300 potential recorded in the MRI field and outside the magnet was not statistically significant, some trend towards a higher amplitude outside, compared to the inside condition could be noticed. The P300 latency was almost identical but the amplitude, as well as the shape of the potential were different. The P300 amplitude can be lowered at its intrinsic generation by various reasons [39]. P300, as an endogenous potential, can be affected by the learning effect [40] and stress [41], in combination with MR conditions and the auditory noise generated by gradient coils. The latter factor might be especially important because it changes the task difficulty [42]. The auditory noise may also be considered as having negative influence on the stimuli quality for the subject, what could cause a reduction in the P300 amplitude [39].

Furthermore, tasks inside and outside the scanner were different in terms of event numbers and randomization. This might also have caused the difference in the P300 amplitude when comparing the two conditions. Several studies have suggested that the P300 amplitude diminishes over the course of an experiment (habituation) [43–45]. Additionally motivation level may influence the amplitude of the P300 wave as well [46]. It is possible that our young subjects might have been slightly more motivated when sitting next to the experimenter than when communicating via intercom during the MRI study. In our opinion it is difficult to determine which of the above factors was essential in the presented study. Therefore, a combination of all mentioned factors should be taken into account.

#### Oddball task

The procedure that was used to randomize the stimuli is atypical for oddball tasks. This decision was driven by the fact that the hemodynamic response needs time to recover to baseline before the next stimulus occurs: the minimum of 9 s is needed between deviant tones to provide at least 90 % BOLD recovery [27]. In addition, our procedure allows for two deviants to occur in a row. Although the P300 potential amplitude might be lowered in response to the second deviant stimulus, it was crucial to prevent loss of attention caused by the long delay between deviants.

There were no significant differences between the latencies of N100 and P300 inside and outside the magnet. However, as mentioned before, in the ERP–fMRI paradigm the P300 amplitude was lower but still evidently different from the standard tone responses.

Another important advantage of the present EEG-fMRI paradigm (Fig. 1) is its short duration of approximately 15 min. Similar experimental protocols reported in literature often take about 30 min [23, 30]. Our participants judged the presented task as not exhausting or monotonous what is important for P300 elicitation. A short paradigm is also advantageous for examining children and clinical populations. ERP registration during continuous fMRI scanning (gradient switching) is still a novel approach. Most papers on the topic presented EEG recordings using sparse fMRI registration, that is in the absence of changes in the magnetic field [30, 47, 48]. In our opinion this approach is controversial and the sound generated by an MRI scanner should be considered as an additional stimulus and, therefore, potentially affecting task performance. Continuous scanning, on the other hand, adds auditory noise which may result in decreased N100 (and P200) responses. The noise is, however, stable during the whole paradigm, and also should not affect the task execution, making P300 responses more reliable.

### Source localization algorithm

We estimated source localization using current density methods [49] rather than dipole fitting for two following reasons: (a) an assumption about the type and number of sources is needed in case of dipoles, (b) correct dipole estimation needs high SNR values. After group averaging our ERPs fulfilled the SNR criterion (SNR = 35) but we still considered the dipole fitting assumptions too arbitrary. In CLARA’s first step a low-resolution electromagnetic tomography (LORETA) is computed [50]. It uses a Laplacian weighted minimum norm algorithm with no a priori assumptions, thus providing a more open solution to the EEG inverse problem, making it more reliable and easier to compare with fMRI data. The CLARA algorithm shares all the benefits of LORETA, but, due to more focal images, allows one to determine the sources more precisely.

### Source localization results

EEG source localization is a mathematical procedure with limited spatial resolution. It is based on discrete 64 electrode locations distanced, in our study, approximately 3 cm from each other. Therefore, it should be considered with caution and one should not expect an exact match between the EEG and fMRI results.

fMRI, due to its good spatial resolution, enabled a precise detection of the following brain regions: middle frontal gyrus, precentral gyrus, inferior parietal lobule, insula, SMA, superior temporal lobe (Fig. 2; Table 1). These areas have been reported to be involved in selective attention [7, 51]. Additionally, we found activity in the thalamus, caudate head and cerebellum. The thalamus engagement is not surprising, as part of neuronal networks underlying emotional, motivational, associative and cognitive functions. It also plays a particular role in attending to auditory stimulation [52]. The caudate head participates in goal-directed actions [53] (in the presented task its activity might have been related to recognizing the stimuli as deviant vs. standard). Cerebellum activity might be related to its involvement in various cognitive functions, such as attention [54].

The LORETA-based source localization revealed fewer sources than fMRI (Fig. 6), what can be explained by biological backgrounds of both signals [19]. BOLD signal corresponds to blood oxygenation changes related to neural activity (mainly presynaptic) and is based on metabolic processes [55], whereas EEG reflects electrical neuronal activity. Importantly, the coverage provided by the EEG technique does not include all brain areas. Due to arrangement of electrodes, the more ventral the source, the less precision is ensured. As an example, the cerebellum cannot be usually monitored with EEG recordings. A second disadvantage of the EEG-based source localization methods is the limitation of deep brain monitoring. Subcortical structures, such as the thalamus cannot be clearly seen with EEG, due to strong dispersion of electrical currents during propagation through tissue. On this basis, it can be assumed that some of the fMRI hot spots will not be correlated with the estimated EEG sources. Although if they are, the relationship can be implied according to the evidence shown by Logothetis et al. [17]. The authors investigated the BOLD signal and intracranial recordings of single-unit, multi-unit activity, and local field potentials (LFPs) in monkeys and showed very high correlations between these two different signal sources. The main goal of simultaneous EEG–fMRI is to shed light on the foundations of the two measures and their interrelations [19]. Considering all the issues described above, we attempt to link source localization results from fMRI and EEG-based CLARA algorithm which have nearby coordinates and are located in the brain cortex.

#### Area A

The source estimated for interval A partially overlapped with fMRI results in the SMA (Fig. 6a). However, we are far from giving any final statements on the brain source location of EEG signal from interval A. For EEG, the SMA is the nearest one to the estimated source but bilateral precentral gyri may also be considered. It is difficult to determine where the signal originates from when its electrical head distribution is mirrored in both hemispheres (compare images presenting scalp distribution starting at 250 and 269 ms in Fig. 5). This issue is known in literature and crucial, especially when determining the localization of the auditory N100 potential [56]. It can also be noticed that CLARA algorithm outcome from interval A was left lateralized. Such result may be a consequence of the inverse problem solution [57]. Measurement of midline sources is affected by hemispheric dipole strength and their directions (depending on the anatomy), which contribute to the global EEG scalp distribution.

Furthermore, one could also say that the EEG voltage distribution in interval A (Fig. 5) might reflect the P200 potential, especially since CLARA results were more posterior than the fMRI results. Nevertheless, since we assured correct stimuli timing in studies inside and outside the scanner, we daresay that if there was P200 present in ERP–fMRI it should have similar latency to this recorded outside the MR room. In addition, P200 is a part of the N100–P200 complex hardly elicited in MRI conditions during our study. Taking it all into account, we propose an interpretation which considers the ERP signal in interval A as the initial part of the P3a component whose source might be located in SMA. A similar location was reported by Rektor et al. [58]. This area seems more probable than the precentral gyri, as it has been found to partake in attention and working memory processes [59–61].

#### Area B

The signal recorded in interval B (Fig. 4) is similar to the P3a waveform presented in the literature (e.g. [5]). The outcome of the CLARA algorithm revealed source localization in the frontal lobe (Fig. 6b). This localization is in accordance with previous studies [1, 5, 10]. However, the conjunction of these outcomes with fMRI results is somehow unclear. Firstly, the increased BOLD signal has been noticed in a few frontal regions, mainly in the bilateral insula and right middle frontal gyrus. However, none of these regions corresponded with CLARA results sufficiently. Therefore, we decided to compare the estimated EEG sources with the BOLD results in the standard versus deviant contrast (i.e. *t* statistic value decrease in deviant to standard contrast). In this comparison we found a relationship with good accuracy between CLARA outcomes and fMRI in the left orbito-frontal cortex (OFC) (Fig. 6b).

There is no unified theory which might explain the decreased BOLD signal in the deviant versus standard contrast and a more obvious explanation could be proposed, such as of positive activity in the reversed condition (i.e. standard to deviant). The OFC recruitment in the reversed contrast might be explained as a habituation effect, as participants were asked to pay attention to deviant tones and ignore standard tones (see [62, 63] for review). However, this theory seems not to be congruent with our EEG data. Our results suggest that the left medial OFC is connected with processes engaged in deviant tone recognition rather than standard tone perception. For standard tone processing inside the magnet no significant electrical response has been found. On this basis, it is very difficult to draw conclusions and we only dare to suggest that processes involved in the generation of the P3a component might occur in left medial OFC. OFC is one of the least understood brain areas, making the interpretation of our results even harder (a comprehensive review of OFC functions can be found in a special issue of Annals of the New York Academy of Sciences vol. 1239, entitled “Critical Contribution of the Orbitofrontal Cortex to Behavior”). It has been proven, however, that OFC is involved in decision-making and response selection [64, 65]. In terms of our study, it could be hypothesized that in response to a deviant stimulus, decision-making on stimulus importance might caused a resources release in OFC resulting in lower BOLD signal. This premise, however, needs further exploration.

#### Area C

The CLARA algorithm, computed for interval C, revealed two mirrored sources depicted in the upper row in Fig. 6c. The ERP response to the deviant tone marked in blue in Fig. 4 corresponded greatly with the template described by J. Polich [5]. The interval can be, therefore, bound with the P3b component. The estimated sources are in line with literature, even in such details as higher signal amplitude on the right side (e.g. [5, 10]). In this interval the correspondence with fMRI results is also very precise. Therefore, we come to a conclusion that P3b originates from bilateral posterior cingulate gyri. Functional roles of these fMRI regions are, among others: attention [51], working memory [61], same-different discrimination (with the advantage of the right hemisphere) [66], integer computation (in the left hemisphere) [66]. All these processes, especially the same-different discrimination, are involved in the oddball task during which the subject is asked to count deviant stimuli differing in frequency. In our study frequency differentiation might be associated with a slightly higher ERP signal amplitude in the right hemisphere (Fig. 4) and resulted in better GLM fitting for the right, compared to the left, inferior parietal lobule (Fig. 3).

#### Correlation between areas

In Fig. 7 a current density (CD) time course for each area revealed by CLARA estimation was shown. Importantly, CD time–amplitude information based on a mathematical solution should not be considered as a biological signal. Additionally, there are noticeable step-like artifacts in CD courses, which seem to result from the CLARA iterative approach. However, the algorithm provides information about correlation between areas and their temporal significance. On this basis it can be assumed that area A is the first activated region. Its withhold point is also a starting point for area B. After approx. 30 ms parietal regions, called area C(L) and area C(R), become engaged and CD amplitudes from these areas start to increase. These three regions are electrically active until 460 ms, when the engagement of area B starts to decline, with electrical activity of areas C(L) and C(R) still increasing. The existence of anatomical connections between OFC (area B) and the parietal lobe (areas C) in cingulum, which was described by Catani and Thiebaut de Schotten [67], can suggest strong functional correlations between these areas. It is noteworthy that cingulum is part of the limbic system, involved in emotion, attention and memory processes.

The presented method of CD time relations between bioelectrical source generators estimated using the CLARA-based algorithm is a novel approach to CD analysis and can provide additional information of brain function. It needs, however, to be verified in further studies and should be optimized to reduce the drawback of CLARA non-linearity. Nevertheless, if this analysis turns out repeatable, it could contribute to a comprehensive theory on the nature of the P300 component, as well as on the relation between brain processes represented by P3a and P3b components.

## Conclusion

The paper presents the results of simultaneous ERP–fMRI study in which a modified oddball paradigm was applied. This paradigm may be used to investigate cognitive processes of children and neuropsychiatric patients. Moreover, it allows for continuous fMRI scanning with whole brain volume coverage that significantly reduce the study time. The CLARA algorithm and an innovative approach to CD time-course analysis, applied for localizing the source of the P300 wave has been also presented. These outcomes may contribute to better understanding of brain activities underling P300 wave and its subcomponents. Therefore, the proposed ERP–fMRI oddball paradigm may be useful for comprehensive investigation of neural correlates of auditory attention.

## Footnotes

1 DLF was the minimum frequency difference between two tones required for their correct discrimination. The DLF test procedure is based on modified oddball paradigm using 20 % target tones and 80 % standard tones. The 100-ms sine wave tones (a standard tone of 750 Hz and a randomly presented target tone of different frequency) were delivered to both ears. The initial frequency difference between standard and a deviant tones was 100 Hz. Participants were asked to press a key when they heard a higher tone compared to standard one.
